# I am Lifted Above the World: utilizing VR for stress reduction among perinatal women of color

**DOI:** 10.3389/fpsyt.2024.1377978

**Published:** 2024-04-23

**Authors:** Judite Blanc, Carolina Scaramutti, Mary Carrasco, Stacyca Dimanche, Laronda Hollimon, Jesse Moore, Rhoda Moise, Vilma Gabbay, Azizi Seixas

**Affiliations:** ^1^Department of Psychiatry & Behavioral Sciences, University of Miami Miller School of Medicine, Miami, FL, United States; ^2^Department of Psychology & the Learning Research Development Center, University of Pittsburgh, Pittsburgh, PA, United States

**Keywords:** women of color, motherhood, perinatal mental health, resilience, virtual reality therapy, intersectionality, Black women, Latina women

## Abstract

**Background:**

Perinatal mental health conditions affect 800,000 individuals annually in the United States and are a leading cause of complications in pregnancy and childbirth. However, the impact of these conditions varies across racial and ethnic groups. Portable digital solutions, such as mobile apps, have been developed for maternal mental health, but they often do not adequately cater to the needs of women of color. To ensure the effectiveness and equity of these interventions, it is crucial to consider the unique experiences of perinatal women from diverse racial backgrounds. This qualitative study aims to explore the complex aspects of motherhood, maternal mental well-being, and resilience among perinatal women of color. It also investigates the factors that either hinder or facilitate the use of Virtual Reality (VR) for stress management in this specific demographic.

**Methods:**

This research involves two focus groups comprising perinatal women, primarily identifying as Black or Latina, enrolled in the ongoing Nurturing Moms study at the University of Miami Miller School of Medicine. Additionally, feedback is collected from five different participants. The study assesses Nurture VR™, a VR-based program integrating mindfulness techniques, relaxation exercises, and guided imagery for pregnancy and postpartum.

**Results:**

Qualitative analysis uncovers five primary themes and 19 sub-themes, addressing the complexities of motherhood, maternal mental health, attitudes towards VR therapy, postpartum care, and the perception of resilience. Participants share challenges related to household management, caregiving, financial stress, breastfeeding, relaxation, sleep, and the significance of social support. Their preferences and reservations regarding VR therapy are also expressed.

**Conclusion:**

This study sheds light on the diverse struggles and obstacles faced by women of color during and after pregnancy, with potential repercussions for their mental and sleep health. It underscores the need for mental health screening and analysis of maternal stress-related sleep issues, in addition to the facilitation of social support in maternal health programs. Additionally, it highlights the promise of culturally responsive behavioral treatments, including VR interventions, in offering timely and tailored mental health support to perinatal women, taking into account their intersectional identities.

## Introduction

Perinatal mental health conditions are leading causes of complications in pregnancy, childbirth, and maternal mortality, and research consistently reveals that approximately 15-20% of pregnant and postpartum individuals in the U.S. experience conditions such as mood or anxiety disorders (1, 2). However, it is crucial to recognize that the impact of the perinatal mental health crisis is not uniform across all racial and ethnic groups. These conditions disproportionately affect women from marginalized communities, highlighting the intersectionality of race, ethnicity, and socio-economic status. For instance, Black women are disproportionately burdened by maternal mental health issues, such as postpartum depression and anxiety, at a rate of 43.9%, compared to their white counterparts at 31.3% (3). Similarly, Latina mothers experience a significantly higher prevalence of depression, with rates ranging from 12 to 59%, as opposed to the 10-15% observed in the general population (4). Mental health issues, particularly depression, are key contributors to maternal mortality and morbidity, with a notable impact in both the United States and Florida (5–7). Importantly, many of these deaths are preventable with proper care and support. Yet, numerous women, especially those from communities of color, face significant barriers to accessing mental health care during the perinatal period, including stigma, a lack of culturally competent care, and disparities in healthcare access (8). This underscores the critical need for culturally tailored interventions to address these disparities and improve access to mental health services, thereby reducing the risk of maternal mortality and enhancing the well-being of mothers across all communities.

### Reframing through an intersectional lens

Minoritized women face compounded stress, shaped not only by their gender but also by their racial identity, experiences that are uniquely intensified for women of color. These stressors, inherently complex and multifaceted, emerge from a blend of various identities, cultural backgrounds, socioeconomic statuses, and other social determinants stressors (1) that perinatal women encounter, as informed by Kimberlé Crenshaw’s seminal intersectionality framework (9). The limitations of traditional psychotherapy, which often overlook gender- and race-specific issues, highlight the critical need for an intersectional approach in addressing maternal mental health. This intricate web of factors heightens the need for interventions that are culturally sensitive and accessible and are meticulously tailored to meet these specific challenges. Such interventions should be designed with a deep understanding of the distinct layers of intersectional stress, ensuring they are responsive to the varied experiences and needs of perinatal women.

To enhance perinatal mental health effectively, a chronological exploration of proposed strategies reveals the field’s evolving understanding and approach. Gibbs pioneers this exploration with an emphasis on empowerment strategies for Black women, addressing the complexities of their intersecting identities and mental health needs, setting an early precedent for intersectional considerations in mental health care (10). Following this foundational work, Grella expands the conversation to include the specific challenges encountered by pregnant and parenting women dealing with both psychiatric and substance abuse disorders, highlighting the nuanced interplay between mental health issues and substance use in marginalized populations (11). Ley et al. further develop targeted interventions with the Healthy Start model, a community-based approach aimed at combating perinatal depression among African American women, acknowledging their unique challenges (12). This initiative underscores the importance of community-focused strategies in addressing maternal mental health. Building on these insights, Gennaro et al. bring attention to the alarmingly high rates of depression and anxiety among racial and ethnic minority women during pregnancy. Their advocacy for comprehensive interventions tailored to meet the diverse needs of these populations emphasizes the critical need for specificity and inclusivity in treatment approaches (13). Adding to the evolution of perinatal care strategies, Singla et al. stress the critical role of patient-centered psychological treatments for perinatal depression, particularly focusing on overcoming barriers to access and the necessity for scalability to ensure these interventions reach a broader audience (14). Concluding with the most recent advancement, McKinney champions the collaborative care model, which seamlessly integrates behavioral health services into primary care settings. This approach effectively treats depression and anxiety across various patient demographics, marking a significant stride towards integrated and accessible mental health care (15).

### The role of digital and virtual interventions

The emergence of digital interventions, particularly Virtual Reality (VR), offers a promising avenue to address pressing issues in maternal mental health by providing accessible and tailored solutions (16, 17). Traditional methods often fall short in addressing the real-time, complex stressors faced by perinatal women of color, necessitating interventions that cater to their unique challenges. While mobile applications have been developed for maternal mental health, they often lack appropriate information and accessibility for women of color, highlighting the importance of considering their specific needs.

Recent studies have shown a growing interest among Black and Hispanic perinatal women in technology-based mental health interventions, emphasizing the need to tailor interventions to their intersectional identities (18). Contrary to previous beliefs, research indicates a significant shift in the utilization of digital mental health platforms among perinatal Black women, with more than half actively using various digital tools for mental health support (19). However, utilization and satisfaction levels vary across different subgroups, underlining the necessity for a nuanced and inclusive approach to digital mental health research.

Innovative approaches like VR interventions hold promise for providing mental health support and relief to perinatal women, particularly in addressing the intersectional challenges they face. VR technology has been shown to alleviate anxiety and pain for pregnant women by offering virtual experiences of the delivery process, acting as a form of safe detachment from immediate surroundings. Despite its potential, data on the efficacy, satisfaction, and lived experiences of VR-based mental health interventions among women of color are limited, underscoring the need for further exploration in this area (20).

Our study seeks to understand the experiences of Black and Hispanic/Latina perinatal mothers, specifically focusing on their use of Virtual Reality (VR) solutions for mental wellness. We aim to measure their satisfaction and engagement and to identify both the obstacles and supportive factors they encounter with VR therapies. Through analyzing their experiences with VR features like breathing exercises, imagery, and videos, this research intends to highlight effective ways to address perinatal mental health challenges. Ultimately, our goal is to inform the development of culturally sensitive interventions that enhance maternal mental wellness among these communities (20).

## Methods

### Study design: Nurture VR™ program

This study was approved by the Institutional Review Board of the University of Miami Miller School of Medicine. All data were de-identified and anonymized. The participants were part of the *Nurturing Moms study (IRB#:20220518)*, a pilot project assessing the efficacy of *Nurture VR™.* The latter is a VR-based maternal health and wellness program designed to support expectant and new mothers through the third trimester, labor, delivery, and postpartum phases. Nurture VR integrates mindfulness techniques, relaxation, and virtual reality-enabled guided imagery, targeting pregnancy and post-partum periods. Participants engaged with the NurtureVR program for five weeks, completing various modules tailored to pregnancy and postpartum stages. The larger Nurturing Moms pilot study goal was to evaluate NurtureVR’s efficacy in reducing stress levels among pregnant and postpartum Black and Latina women.

### Nurturing moms study selection criteria

The study utilized specific inclusion and exclusion criteria to select participants. Inclusion criteria encompass individuals aged 18 and above, English speaking, identifying as Black or Latina, and being pregnant (up to 36 weeks) or postpartum (up to 12 months after birth). Exclusion criteria encompassed individuals not identifying as Black or Latina, not pregnant or postpartum, not speaking or understanding English, self-reported diagnosis of psychosis, a history of seizure or vertigo, significant vision or hearing impairment, and a history of significant motion sickness, epilepsy, or sensitivity to flashing light/motion or injury to the eyes/face/neck.

### Nurturing moms’s qualitative phase recruitment and participants

#### Semi-structured interviews and focus groups

Participants for the focus group were selected through convenience sampling techniques from a cohort that satisfied the inclusion criteria and had been exposed to the Nurture VR program. The age distribution of participants ranged from 25 to 42 years. The demographic composition of the sample was notably diverse, with 40% identifying as Black, 30% as Hispanic, 20% as White Hispanic, and the remaining 10% reporting other ethnic affiliations. Linguistic diversity was also present within the sample; the predominant language spoken at home was English, though variations were observed, including one household speaking Haitian Creole, one a combination of English and Haitian Creole, and another Spanish. Educational backgrounds among participants varied between associate degree (n=2), bachelor’s degree (n=5), or having pursued some form of graduate or professional education (n=1). Employment status showed that a significant number of participants (n=6) were not employed, preferring to identify as housewives. Religiously, a majority declared adherence to Christianity (n=6), while the remainder exhibited a range of beliefs encompassing atheism, non-religiosity, and various other spiritual orientations.

After obtaining written consent, two focus groups were conducted, each consisting of five participants. These sessions were facilitated by a trilingual (English, French and Creole speaker) assistant professor with a doctoral degree in clinical psychology and was supported by English-Spanish bilingual study staff members with qualitative interviewing and experience working with similar populations. The focus groups were conducted via videoconference, each lasting approximately 60 to 80 minutes, and were recorded for accuracy. Subsequently, videoconference recordings were transcribed verbatim by an external transcription company to ensure precise documentation of the discussions. Participants were informed that the discussions within the focus groups were confidential, and any information shared would be privileged solely to the research team approved by the IRB. They were actively encouraged to engage in the discussions to the extent they felt comfortable, with a clear assurance that it was entirely acceptable if there were any questions they preferred not to answer.

The methodology for the focus group discussions employed open-ended questions, permitting participants to elaborate on their experiences and viewpoints with minimal restriction. The interview guide was meticulously designed to encompass a range of subjects pertinent to maternal care. These subjects included the daily challenges encountered by mothers and expectant mothers, their methods for alleviating stress, barriers to accessing quality mental health services, and elements contributing to resilience. Additionally, the guide delved into participants’ natural coping mechanisms, their perception of pharmacological treatments for perinatal mental health issues and overall well-being, as well as their involvement with embodied therapy practices. The selection of these topics aimed to evaluate the participants’ knowledge and attitudes towards these crucial aspects of maternal health. **Table 1** delineates the thematic domain and corresponding exemplar interview questions.

**Table 1:** Focus Group Interview Guide.

Domain	Questions
Navigating Motherhood and Maternal Stress	Can you share your personal challenges and experiences with motherhood? How do you think these struggles have impacted your overall health?
Maternal Mental Wellness	What experience do you have with mental health care? Have you experienced any barriers accessing mental health care?
Stress Management and Self Care	What are things you do to relieve stress? How does stress affect your day-to-day?
Postpartum Experiences and Resilience	The idea of being strong and handling tough times in your life. What does that mean to you?
Embodied Therapy/Nurture VR Experience	Can you share your thoughts on using VR to manage stress? How has this been helpful/unhelpful to you? What are some of the challenges of VR?

Throughout the focus group discussions, participants were encouraged to share their experiences related to the utilization of the Nurture VR program, including their perceptions and the obstacles faced in employing VR technology for maternal stress management. This discourse extended to detailing the challenges they faced during pregnancy or in their roles as new mothers, in addition to discussing their strategies for navigating stress and mental health concerns. The sessions were intentionally structured to facilitate a guided yet open dialogue, enabling a thorough exploration of each participant’s experiences and insights, thereby enriching the qualitative data collected on the efficacy and user experience of the Nurture VR program in a maternal health context.

### Procedure and data analysis

In our study, we employed a general inductive approach for our focus groups and qualitative analysis, focusing on the experiences of participants with VR therapies. By carefully analyzing transcribed interviews, we aimed to delve into the daily struggles and support mechanisms related to maternal mental health care. This method allowed us to assess participant satisfaction and engagement with VR features such as breathing exercises, imagery, and videos. Our objective was to identify barriers and facilitators within these experiences, guiding the development of culturally sensitive interventions to improve maternal mental wellness among these communities. A general inductive approach is commonly used in the health and social sciences and allows findings to emerge from the most frequent and dominant codes and themes encountered throughout the analysis (21). The authors were guided by the interview transcripts to derive the concepts, themes, and interpretations of the objectives.

Following a general inductive approach, the team read both focus group transcripts. A codebook was created that included code names, definitions, sample quotes, and coding decision rules. The approach entailed data exploration, inductive coding, and thematic analysis. Transcripts were read, re-read, and coded in an iterative fashion. An initial list of themes and subthemes was created. Data saturation was met after the second focus group when no new themes or sub-themes emerged for this particular population. Team members then coded one transcript together, discussing coding discrepancies, and adjusting coding decision rules and definitions accordingly. The percentage agreement was calculated to ensure interrater reliability of the codebook. In order to uphold the integrity and credibility of the data analysis process, rigorous quality assurance measures were implemented. Initially, to mitigate any inconsistencies in the thematic analysis, the research team engaged in rigorous deliberative sessions aimed at reconciling coding discrepancies. This process was essential for maintaining the integrity and reliability of the data interpretation, ensuring that all thematic categorizations were consistently applied across the dataset. Through these collaborative discussions, the team was able to achieve a unified understanding and application of the coding framework, thereby enhancing the methodological rigor of the qualitative analysis. Additionally, external validation was sought by soliciting feedback from subject matter experts, providing an independent perspective to validate the interpretations drawn from the data. These multifaceted approaches not only served to mitigate potential biases but also bolstered the robustness and reliability of the study’s conclusions, ensuring that the research outcomes accurately reflected the lived experiences and perspectives of the participants.

All final codes were analyzed in Nvivo (Lumivero, Version 14) (22). Specifically, code frequencies were extracted, allowing identification of the most highly endorsed codes in the data. Then, the information was extracted on code-cooccurrence to understand the number of times two or more codes appeared together in the same excerpt. Lastly, excerpts from each code were discussed to validate the data and codes and gather sample quotations for our themes and subthemes.

## Results

**Figure 1:**
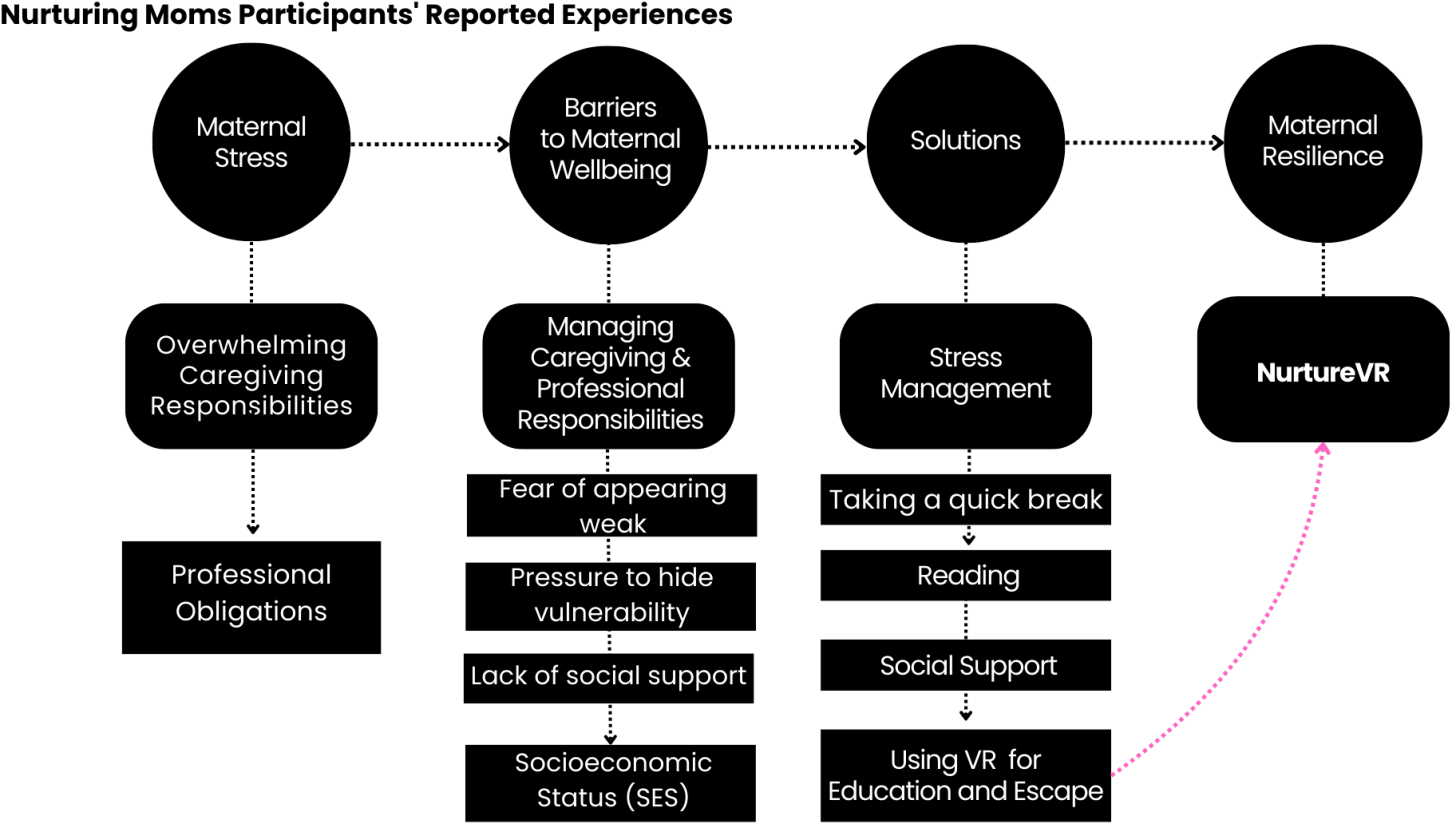
Experiences and perceptions of mothers on motherhood and embodied therapy.

We analyzed focus group data from ten individuals. Age ranged from 25 years of age to 42 years of age (with a mean age of 32.5 years old). All participants identified as female and were enrolled in the ongoing Nurturing Moms pilot study (**Table 2**). Our qualitative analysis revealed five primary themes, each encompassing a nuanced array of 19 sub-themes. The thorough analysis identified a total of 94 references to themes and sub-themes within the qualitative data. **Figure 1** provides an overview of the themes and subthemes that emerged.

**Table 2:** Participant Demographic Profile.

Sample Characteristics	n
Age	
(mean, range)	32.5 (25-42)
ETHNICITY	
White	2
African American/Black	4
Hispanic	3
Other	1
Hispanic Origin	
Yes	5
No	5
Place of Birth	
USA	4
Dominican Republic	1
Haiti	2
Puerto Rico	1
Honduras	1
Nigeria	1
Language Spoken at Home	
English	7
Haitian Creole	1
English and Haitian Creole	1
Spanish	1
Religious Affiliation	
Christianity	6
Atheist/Agnostic	1
No Religious Affiliation	2
Other	1
Marital Status	
Married/Living with a Partner	6
Divorced	1
Single	2
Separated	1
Education	
High School/GED	1
Technical School Certificate	1
Associate Degree	2
Bachelor’s Degree	5
Graduate or Professional School	1
Employment	
Yes	4
No	6
Income	
<$10,000	1
$10,000 - $19,999	3
$20,000 - $39,999	1
$40,000 - $59,999	1
$60,000 - $100,000	2
>$100,000	2

### Navigating motherhood and maternal stress

Motherhood, with its myriad intricacies, presents many challenges, including consistently identified time management difficulties. This challenge extends across various dimensions, encompassing the delicate balance of allocating time equitably among children and a spouse, safeguarding moments for self-care, managing household chores, securing adequate sleep, and meeting work-related commitments.

*“One thing I struggle with is time management, I guess before it would be like I can just get up and go anywhere and now I barely have time to take care of myself.” – Participant 1, expectant mother, African American.*

*“I ask myself, am I going to have time to eat, shower and be human as well?” – Participant 4, mother of two, Puerto Rican.*

*“So I think personally for me, that is another struggle because I feel in 2023 I’ve seen women do many things that is being a mom, that’s working while being a mom, that’s having your kid on your lap while you’re holding a meeting because your kid is fussy and you’re trying to calm them down, that’s multitasking.” – Participant 3 is a Haitian-American woman who joined the study at baseline while pregnant. She participated in the focus group after having her baby.*

*“While I was on my maternity leave, I think I was not doing anything to cope with stress, but I also was not really stressed besides just being sleep deprived and wanting to sleep … I was happy during that period. But when going back to work and then like resuming my life before I became a mom. That’s when I think I started experiencing stress.” –Participant 3, mother of one, Haitian-American.*

The motherhood journey is further complicated by bodily changes and the introduction of new responsibilities, such as breastfeeding, prompting mothers to grapple with the logistics of when and where these activities can be seamlessly integrated into their schedules.

*“I struggle with breastfeeding in public. Like my struggle is finding a time to do it before I leave the home or while I am out finding an area where people are not staring at me. I feel it’s a bit of a mess there. I also have two kids and when we are out, it is chaotic when I have to breastfeed and also pay attention to my other two.” – Participant 2, mother of three, Honduran.*

Noteworthy in this intricate navigation is the commendable support from spouses or partners who share in this profound journey. Many participants expressed profound gratitude for this collaborative effort, recognizing the value of having a supportive companion. However, it’s crucial to acknowledge that, despite this assistance, the overarching responsibility for managing most tasks still falls upon the mother, making the assistance, albeit appreciated, inherently limited in its scope. In essence, mothers find themselves at the helm of the myriad responsibilities that come with motherhood, necessitating adept time management strategies to navigate this complex terrain.

*“Once my husband would get home, it would become a little easier with each of us having one child. What I would try to do is switch the kids. I have him take care of the baby and I spend more time with my daughter because I want her to know she is as equally important. I am trying to do everything and anything house related when my husband has the kids where I can fold clothes, clean, or just take notes of what is needed in the house.” – Participant 4, mother of two, Puerto Rican.*

### Maternal mental wellness

Maternal well-being is intricately tied to the accessibility and timing of care. Among the participants, a common theme emerged – the desire for meaningful connections and a supportive network during their motherhood journey. Many expressed a longing for someone with whom they could share their thoughts and experiences, seeking a connection that transcends mere conversation. Some found solace in connecting with therapists who had undergone similar experiences, appreciating the authenticity and relatability that comes with shared understanding.

*“I’m currently going to see a therapist. I find that it’s been helpful to just talk it out. It really depends on who the person is that you’re talking to. My first therapist talked to me like a friend. I could tell her all my anxiety. I feel a connection is more important than a questionnaire.” – Participant 5, expectant mother of one, Hispanic.*

The essence of human-to-human contact was underscored as indispensable in the context of the motherhood journey, often described as a solitary path marked by transformative changes in both body and mind. Mothers, feeling the weight of this transformative journey, stressed the importance of being heard. The need for a safe space to freely express their emotions and “vent” emerged as a recurring theme, with an acknowledgment of the inherent guilt that often accompanies the act of unburdening oneself.

In summary, the maternal well-being narrative revealed a collective yearning for connections that go beyond surface-level interactions. These women recognized the power of shared experiences and empathetic, genuine connections, emphasizing the crucial role of emotional support and non-judgmental spaces in navigating the complexities of motherhood.

### Stress management and self-care

Women consistently emphasized the profound influence that even a mere five minutes of a break can have on their effectiveness as mothers. Delving into the nuances of their experiences, many detailed how seemingly small activities exert a significant impact on their overall demeanor and well-being throughout the day. Moments of respite, such as listening to music, stealing a few minutes of solitude, or taking a brisk walk, were identified as transformative practices.

*“My fiancé, my mom, my sister, someone, even if it’s just for a couple of minutes, if I’m home alone all day with her, once someone does come over or whatnot, just a couple minutes to be by myself, or like I said go to the gym for an hour and just be by myself, then I can come back a brand new person.” – Participant 1, expectant mother, African American.*

*“Even if it’s five to ten minutes to take a breath, one of my main things is I really like to move and it’s nice to just put music on, put your headphones in and move your body. I feel you come back a brand-new person.” – Participant 1, expectant mother, African American.*

While some mothers explored the therapeutic benefits of physical exercise outside the home, a prevalent theme emerged - the pervasive sense of “mom guilt” associated with momentarily stepping away from their infants. The struggle to balance self-care with the responsibilities of motherhood was palpable, with these women navigating a delicate equilibrium.

“*I’ve always been a big reader but when I decide to do these things, I get the worst mom guilt. And I guess it’s because my own mother wasn’t present. I feel taking an hour to go to the gym or going on a walk makes me feel I don’t want to be around them, and I hate that feeling.” – Participant 4, mother of two, Puerto Rican.*

*Social support*, deemed crucial in mitigating the inherent loneliness of the motherhood journey, was underscored. Participants fondly reminisced about the freedom they once had to socialize with friends, highlighting the stark contrast to their current reality. Additionally, the consensus among the women reflected a poignant acknowledgment of the scarcity of time, lamenting the challenge of incorporating even the simplest stress management tasks into their demanding schedules.

### Resilience

Cultural variances exert a profound influence on the perception of resilience, particularly as articulated by the resilient women in minority communities. For these women, resilience is not merely a quality but a defining aspect of their identity. They articulate a collective ethos of pushing forward and embodying strength, attributes ingrained through generations of cultural wisdom. The women candidly discuss prevailing stereotypes associated with their cultural backgrounds, encompassing the robust expectations placed on Black and Hispanic women. Within this context, the concept of strength is not a mere suggestion but an inherent obligation.

*“Everywhere as a Black woman, you have to be strong and I just can’t stand to hear that because it’s like why are we the only race that have to be strong? Why everybody else gets to cry, get to show emotion, get to feel, but we always have to be strong.” – Participant 6, expectant mother, Haitian-born.*

These women embrace resilience not as a choice, but as an imperative dictated by the unyielding demands of their roles as mothers. They illuminate the intricate connection between motherhood and resilience, asserting that the very act of being a mother compels them to embody this strength. In essence, their narratives unveil a profound interplay between cultural expectations, generational wisdom, and the intrinsic resilience demanded by the profound responsibility of nurturing and safeguarding their children.

*“I was going through a divorce and two months later I found out I was pregnant. I choose to keep the baby. And some stuff like we went through during our young age and now while being pregnant, everything come back. It’s just like I thought we forget about these things. Why are they coming back right now? We need the help, we need the support but I didn’t have that. I had to be strong even though I don’t like the word strong but I had to be strong. It was a must.” – Participant 8, expectant mother of one, Haitian born.*

*“The idea that you just got to keep pushing forward even if the day is tough, like you have to. You’re a mom, you got to like suck it up and do dues, because these kids need you, and then at the end of the day, when you want to unwind and go to bed, you end up scrolling through your phone, because that’s your only me time.” – Participant 5, expectant mother of one, Hispanic.*

### Postpartum experiences

Numerous participants shared poignant narratives about postpartum experiences as an integral facet of the motherhood journey. A prevailing theme emerged as these mothers reflected on the need to project a resilient exterior, concealing their inner struggles from both partners and children while silently navigating the challenges. The discourse extended to the indispensable role of *medication* in alleviating the burdensome symptoms of postpartum, as these manifestations posed obstacles to fulfilling their roles as effective parents.

*“I don’t want to say I don’t think they understand, but I think I want to say that they don’t understand because they don’t understand what’s going on with our bodies, the hormones, our own expectations or trying to in my case not be my mother and be a better mother.” – Participant 4, mother of two, Puerto Rican.*

Acknowledging the imperative of addressing postpartum challenges, participants advocated for innovative approaches, including the exploration of new medications for postpartum depression (PPD) that offer swift relief from symptoms. Their openness to alternative solutions underscored a collective commitment to fostering an environment that supports maternal well-being and facilitates more effective parenting.

“Regarding the medication, the type of insurance that I have at the moment is just a discount and they don’t really cover specialist. I do pay out of pocket for my psychiatrist … I would be interested in trying out the medication because I do feel that my postpartum is intense.” – *Participant 4, mother of two, Puerto Rican.*

*“Two weeks after I had my baby, I had preeclampsia and my baby is not even a month yet. So I’m still dealing with it. If I can get my hand on it, it will be helpful to me.”* – *Participant 8, mother of one, Haitian born.*

*“I like to go to like using the VR headset first before I try medicine or doing like therapy first before I try medicine, and I think to me like doing those process first before you resort to medicine is very important. That’s like my virtue, but I do like that there is something available. I think it’s necessary and it helps to show society that yes, moms need help and I can’t believe it’s taken this long.” – Participant 5, mother of one, Hispanic.*

### Embodied therapy

The participants in these focus groups conveyed positive sentiments regarding embodied therapy, particularly expressing favorable feedback on the Nurturing Moms Virtual Reality program. Their comments spanned the spectrum, with many highlighting the immersive experience that allowed them to “escape” into guided imagery and relaxation techniques. Additionally, participants voiced appreciation for the engaging and beneficial nature of the videos incorporated into the virtual reality sessions.

*“It is very educational. I think by the time that I was part of the study, I already had some other outlets. So some like apps on my phone that was already walking me through every development stage of the baby weekly. But yeah, seeing it on the VR was different. Like I said, when I was using it the couple of times I used it, I felt like I was lifted above the earth.” – Participant 3, mother of one, Haitian-American.*

*“It is really educational and relaxing some of the things on it. It was really different.” – Participant 2, mother of three, Honduran.*

*“I found it educational as well. It helped me learn a lot, especially about breastfeeding. I like those segments on that and it definitely did make me feel a lot more prepared for labor and having a child.” – Participant 1, expectant mother, African American.*

However, a notable subset of participants shared concerns about the inability to multitask while using the virtual reality headset, underscoring the essential nature of multitasking in their roles as mothers. This limitation resonated with the broader challenge of finding precious moments for self-care within their demanding schedules.

*“It is like a blessing in a way, because I think as a mom, you’re built to multitask, like you’re built to carry a baby and do laundry and cook dinner and whatnot. But with this, you can’t.”* – *Participant 1, expectant mother, African American.*

*“I need to be in the presence of a human being and I can’t get that connection with any headset or device.” – Participant 4, mother of two, Puerto Rican.*

*“You can do it at 12:00 PM. You can do it at 12:00 AM. It was just like, okay I need to find at least an hour to sit down and have no distractions and do it. But again, once you start it and you start learning and going through the experience, it is a great experience.” – Participant 1, expectant mother, African American.*

In the study, in addition to the semi-structured interview portion, five different mothers provided written feedback to our focus group feedback questionnaire. Their ages ranged from 24 to 36 years, with a mean age of 29. All respondents who filled out the feedback form were females and enrolled in the Nurturing Moms VR pilot study. Among these participants, two were first-time mothers, while three had previous children. The sample was nearly evenly divided in terms of employment status: three mothers were employed, and two were stay-at-home mothers.

In similar feedback to our focus groups, these five mothers’ written insights highlighted the difficulty of coping with physical and emotional changes during pregnancy, the challenges of accessing mental health care, coping strategies for stress, and the potential benefits of VR therapy in managing stress. Some noted the importance of addressing postpartum mental health, while others expressed initial skepticism about VR’s effectiveness but later found it helpful (See **Table 3**).

**Table 3:** Summary of additional written feedback received from nurturing moms study participants.

Domain	Question	Quotes
Struggles in Motherhood or Mother-to-Be	Please share your perspective on the particular struggles and stressors you’ve encountered as a new mom or pregnant person? Are there any aspects that you believe are often overlooked or not fully understood by others? Please share specific challenges or stressors you believe Black or Hispanic moms or pregnant individuals face that may not be widely recognized or understood in healthcare and research?	“As a pregnant woman, our body and hormones and emotions change. Physically and emotionally, we must prepare to bring forth life despite struggles and stressors. Most people overlook this and believe it is easy to cope with a new baby on the way.” – *Written Focus Group Participant 5, mother of one, Hispanic, Stay-at-home mom* “I believe in the Hispanic Culture postpartum depression is often overlooked. Especially older generations believe it is a lie. Oftentimes making it difficult for moms to feel understood.” – *Written Focus Group Participant 2, mother of one, Hispanic, Working-mother*
Mental Health Care	Please provide your opinion on barriers to accessing quality mental care for mothers or pregnant people. Share any challenges you’ve faced in obtaining mental health therapy, especially considering any unique barriers related to your background.	“Mental Health is oftentimes seen in a very negative light causing moms to not want to seek help.” – *Written Focus Group participant 2, mother of one, Hispanic, Working-mother* “I have to pay for a psychologist since my insurance does not cover it completely and I believe that postpartum mental health is as important as during childbirth.” – *Written Focus Group participant 4, mother of one, Hispanic, Working-mother*
Stress Management	Share any feelings you have about coping with stress. Describe what makes you stressed, and also provide your opinion on using VR for stress relief.	“I do not personally cope with stress well, but I do try to sleep on it and look at situations from all perspectives to calm myself when I feel I am beginning to get anxious. Try to figure out where it stems from.” – *Written Focus Group participant 1, mother of two, Hispanic, Working-mother* “During my time with the VR set I noticed it kept me occupied and provided useful resources to inform me on my pregnancy.” – *Written Focus Group participant 5, mother of one, Hispanic, Stay-at-home mom*
Resilience	Share any feelings you have about resilience in motherhood. Please share what is helpful for you to stay resilient in the face of stress. Discuss any strategies that have helped you handle tough times.	“I have no other choice I have three littles that depend on me even when I’m falling apart.” – *Written Focus Group participant 3, mother of three, Hispanic, stay-at-home mom*
Nurturing Mothers Virtual Reality (VR)	Share any feelings you have about VR (virtual reality) for stress relief. Describe any prior VR experiences, your initial perceptions of using VR for stress management, and whether you’d recommend it to others in your community.	“It has helped but still doesn’t help 100% as a mom of three you are always trying to make sure everyone is happy and healthy and with so many sickness.” – *Written Focus Group participant 3, mother of three, Hispanic, Stay-at-home mom* “At first, I didn’t believe the VR set could reduce my stress but as I used it I sensed my worries go away. I was enjoying my time with the VR and all the tools and resources provided. I would recommend this for stress management.” – *Written Focus Group participant 5, mother of one, Hispanic, Stay-at-home mom*

## Discussion

To address the critical issue of maternal mortality, which disproportionately impacts perinatal women of color, experts have highlighted the need to enhance maternal mental health as a pivotal strategy. This approach is essential not only for the well-being of mothers but also for their infants. In this endeavor, Virtual Reality (VR) therapy presents itself as an innovative and promising tool. It stands out for its ability to circumvent traditional barriers in mental health care, offering an approach that is acutely attuned to the needs of this specific demographic. VR therapy offers a range of in-demand, dynamic, standardized, and embodied digitally facilitated techniques, including immersive breathing exercises, guided imagery, and tailor-made audiovisual stimuli (20). Virtual support can be especially beneficial for perinatal women who face higher levels of anxiety and stress due to their intersectional identities, such as their racial and gender identities. By offering a safe and controlled environment for acclimation, VR can help mitigate the anxiety and pain associated with childbirth and postpartum. VR can act as a distraction, allowing women to focus beyond their immediate challenging surroundings. This distraction is particularly important when considering the unique stressors that perinatal women from minoritized backgrounds may experience. Whether the concerns are related to pregnancy, social support, healthcare accessibility, discrimination, or socio-economic challenges, VR can provide a valuable respite from these stressors during a critical period (20).

This qualitative study aimed to explore the complex experience of motherhood and its impact on the mental well-being and resilience of perinatal women of color in relation to VR therapy as a portable digital health intervention. Specifically, we analyzed qualitative data from two focus groups and written feedback from Black and Latina women to gain a comprehensive understanding of participants’ experiences and perspectives. The results yielded narrative insights across five primary themes: 1) Navigating Motherhood, 2) Maternal Mental Wellness, 3) Resilience, 4) Embodied Therapy, 5) Postpartum.

### Nurturing mother’s virtual reality experience

The findings from our qualitative study provide a comprehensive and nuanced perspective on perinatal mothers of color engaging in embodied therapy for stress management. Many participants expressed overwhelmingly positive sentiments, indicating that the immersive nature of VR effectively engaged them, underscoring its efficacy in cultivating a unique experience through guided imagery and relaxation techniques.

A notable theme that emerged was the participants’ *sense of escapism*, exemplified by Participant 3’s feeling of being “lifted above the world.” This sense of detachment from their current environment suggests that VR can not only inform but also create a positive and stress-relieving atmosphere for perinatal mothers as needed. This aspect holds particular value, given the emotional and psychological dimensions associated with the journey of pregnancy, childbirth and postpartum. Participants commonly appreciated the relaxing nature and educational content of VR, particularly its effectiveness in preparing them for labor and providing insightful information on motherhood. Participant 1’s endorsement of these unique features further supports VR’s potential to offer an experiential learning environment beyond traditional educational methods.

However, this discussion also highlights a subset of participants who expressed concerns regarding the drawbacks associated with virtual reality. Notably, the inability to multitask while using a VR headset was identified as a significant disadvantage. Given the challenges faced by expectant and/or new mothers in balancing their roles, this concern highlights potential difficulties when incorporating VR into their daily lives. This issue underscores the need to develop new strategies and solutions to integrate VR effectively into their daily responsibilities. Shorter, focused sessions may address this issue and enhance their overall experience.

Furthermore, the reported adjustment period required for VR use and the absence of human interaction underscores the importance of a balanced approach when incorporating digital applications into maternal health education (19). While VR can offer personalized and engaging learning experiences, the value of human interaction and support should not be underestimated, especially when addressing emotionally charged topics related to motherhood. The integration of virtual reality with real-time interaction or support systems presents an opportunity to enhance the overall experience, making it more comprehensive and practical. Despite these reservations from participants, acknowledging both the challenges and advantages of virtual reality suggests its supplemental value and beneficial addition to their self-care toolkit.

This qualitative study sheds light on diverse and dynamic maternal experiences with embodied therapy, particularly within the realm of virtual reality. The positive feedback indicates that these interventions have the potential to improve maternal well-being. However, the reported drawbacks emphasize the importance of considering practicality and adaptability when designing therapeutic digital solutions for perinatal women. Further research is warranted to gain a deeper understanding of these dynamics and to enhance virtual reality programs to better suit the needs of perinatal women who seek effective and accessible self-care interventions.

### Barriers to accessing care, postpartum, and resilience

Understanding the challenges related to accessing care for perinatal women of color is paramount. Our findings reveal that, during the postpartum period, participants encountered community and systemic barriers that hindered their access to behavioral treatments and maternal health services. Issues such as cultural barriers and cultural incompetency, the superwoman schema (23), gender roles, caregiver burden, unaffordable mental health services, unfriendly workplace policy, and economic constraints were noted. Additionally, the postpartum period itself presented unique stressors, making it challenging for some participants to engage fully with the VR program.

A unique quality of our collected population was the equal distribution of stay-at-home or unemployed mothers and working mothers. Our population highlighted and emphasized in the qualitative data the perceived necessity for social support and connectivity. Prior research has shown that stay-at-home or unemployed mothers feel less connected and receive poorer protective effects of social support than working mothers (24).

Future research on digital health interventions for perinatal stress and resilience may leverage these insights to incorporate social connectivity and networks in black and brown populations.

The postpartum phase represents a critical time for maternal mental health support, highlighting the need for holistic and tailored interventions that consider the specific needs and vulnerabilities of perinatal women of color during this transitional period.

### In demand VR-based therapy and resilience building

In terms of resilience, our study offers insights into how VR therapy can contribute to building resilience among perinatal women of color. Participants reported increased self-awareness, emotional regulation, and coping skills as outcomes of their engagement with VR-based stress reduction programs. For instance, Participant 4’s feedback highlights the flexibility, challenges, and positive outcomes associated with participating in the Nurturing Moms study, offering valuable insights into the experience of mothers in similar programs.

These insights suggest that VR therapy has the potential not only to alleviate maternal stress but also to empower perinatal women of color with tools to navigate the stressors of motherhood. Recognizing and enhancing resilience is crucial for promoting mental well-being and long-term positive outcomes for both mothers and their infants.

### Limitations/strengths

This study is not without limitations. Firstly, the qualitative nature of our research limits the generalizability of our findings. While the in-depth exploration of participants’ experiences provided valuable insights, the sample size was relatively small, and the participants were predominantly Black and Latina women, mostly from the South Florida region. Thus, caution should be exercised when extrapolating our results to broader populations. Furthermore, the broad acceptance of VR therapy is hindered by challenges related to cost-effectiveness, inclusivity, and cultural appropriateness, particularly for individuals from lower socioeconomic backgrounds (25). Stigma also poses a substantial barrier to integrating VR therapy into maternal care. Despite the increasing recognition of digital health tools, societal stigma persists around the utilization of technology-based interventions for health and well-being, VR included. This stigma can take various forms, such as the perception of escapism, concerns over technology dependence, and the stigma surrounding mental health. Addressing these challenges requires concerted research efforts aimed at developing virtual care programs that improve accessibility and engagement for socioeconomically disadvantaged groups. This includes populations with limited access to the internet or telecommunication services (1).

## Conclusion

However, these limitations are balanced by notable strengths. The qualitative approach allowed us to delve deeply into the experiences and perspectives of perinatal women of color, providing rich and nuanced data. Moreover, our study’s focus on intersectionality and the use of VR therapy as a digital intervention in the context of maternal mental health represents novel and innovative contributions to the field. The potential of VR technologies in alleviating stress among pregnant and postpartum women, particularly during childbirth, demonstrates promise (26). Our findings lay the foundation for future research that can further explore these themes and refine digital interventions to cater to the unique needs of perinatal women of color that are overly burdened by the maternal health crisis. Overall, this study serves as a valuable starting point for addressing maternal mental health disparities and promoting well-being among perinatal women of color through innovative means.

## Data availability statement

The data presented in this article are not publicly available due to IRB restriction. Requests to access the data should be directed to the corresponding authors.

## Ethics statement

The studies involving humans were approved by University of Miami Miller School IRB. The studies were conducted in accordance with the local legislation and institutional requirements. Written informed consent for participation in this study was provided by the participants’ legal guardians/next of kin. Written informed consent was obtained from the individual(s), and minor(s)’ legal guardian/next of kin, for the publication of any potentially identifiable images or data included in this article.

## Author contributions

JB: Writing – review & editing, Writing – original draft, Visualization, Supervision, Software, Resources, Project administration, Methodology, Investigation, Funding acquisition, Formal analysis, Data curation, Conceptualization. CS: Writing – original draft, Methodology, Formal analysis. MC: Writing – original draft, Project administration, Data curation. SD: Data curation, Writing – original draft, Project administration. LH: Writing – original draft, Software, Data curation. JM: Writing – review & editing, Software, Project administration, Data curation. RM: Writing – review & editing. VG: Writing – review & editing. AS: Writing – review & editing, Validation, Supervision, Methodology, Funding acquisition, Conceptualization.
